# Understanding the hydrological response of a headwater-dominated catchment by analysis of distributed surface–subsurface interactions

**DOI:** 10.1038/s41598-023-31925-w

**Published:** 2023-03-22

**Authors:** Ilhan Özgen-Xian, Sergi Molins, Rachel M. Johnson, Zexuan Xu, Dipankar Dwivedi, Ralf Loritz, Utkarsh Mital, Craig Ulrich, Qina Yan, Carl I. Steefel

**Affiliations:** 1 Earth and Environmental Sciences Area, Lawrence Berkeley National Laboratory, Berkeley, USA; 2 Institute of Geoecology, Technische Universität Braunschweig, Brunswick, Germany; 3 Institute of Water and River Basin Management, Karlsruhe Institute of Technology, Karlsruhe, Germany

**Keywords:** Environmental sciences, Hydrology

## Abstract

We computationally explore the relationship between surface–subsurface exchange and hydrological response in a headwater-dominated high elevation, mountainous catchment in East River Watershed, Colorado, USA. In order to isolate the effect of surface–subsurface exchange on the hydrological response, we compare three model variations that differ only in soil permeability. Traditional methods of hydrograph analysis that have been developed for headwater catchments may fail to properly characterize catchments, where catchment response is tightly coupled to headwater inflow. Analyzing the spatially distributed hydrological response of such catchments gives additional information on the catchment functioning. Thus, we compute hydrographs, hydrological indices, and spatio-temporal distributions of hydrological variables. The indices and distributions are then linked to the hydrograph at the outlet of the catchment. Our results show that changes in the surface–subsurface exchange fluxes trigger different flow regimes, connectivity dynamics, and runoff generation mechanisms inside the catchment, and hence, affect the distributed hydrological response. Further, changes in surface–subsurface exchange rates lead to a nonlinear change in the degree of connectivity—quantified through the number of disconnected clusters of ponding water—in the catchment. Although the runoff formation in the catchment changes significantly, these changes do not significantly alter the aggregated streamflow hydrograph. This hints at a crucial gap in our ability to infer catchment function from aggregated signatures. We show that while these changes in distributed hydrological response may not always be observable through aggregated hydrological signatures, they can be quantified through the use of indices of connectivity.

## Introduction

Runoff generation and overland flow results from the interactions among rainfall, surface topography and roughness, and soil physical and hydrological properties^1^. These interactions control the development of preferential flow paths both on the surface and in the subsurface, which route net water fluxes towards the catchment’s outlet^2,3^. The in- and exfiltration processes along these preferential flow paths result in transient spatial patterns of hydrological state variables^4^. Runoff formation processes occur at small spatio-temporal scales (submeter, minutes), but manifest themselves in hydrological signatures across scales up to the catchment scale. The mechanisms of this *flux re-scaling* are still not completely understood^5,6^.

Runoff formation and preferential flow paths are interlinked with other environmental processes^7^. For example, increased infiltration along preferential flow paths creates local pressure gradients, concentration gradients or depletion of soil moisture, biogeochemical hotspots^8^, and vegetation patterns in water-limited ecosystems^9^. Further, spatial dynamics of infiltration and exfiltration, controlled by the interactions between surface and subsurface, regulate the runoff generation and formation in the catchment itself. Thus, understanding the development of runoff formation and preferential flow paths can give insight into spatial patterns of hydrology-driven biogeochemical and ecological processes^10,11^.

Most runoff modeling studies consider catchments with no significant inflow from upstream areas^1,12–18^. In contrast, in catchments that receive upstream water, the hydrograph may be controlled by this inflow from their headwaters. A common challenge is that the headwater inflow “drowns out” the catchment’s own response. In such *headwater-dominated catchments*, distinct states of runoff formation cannot be observed in the aggregated hydrograph. This makes it difficult to identify the hierarchy of controls on the runoff formation. From a modeling perspective, these challenges manifest themselves during model calibration, because the dominance of the boundary condition makes it difficult to assess whether the system has been properly parameterized. It can be argued that these challenges arise because we are essentially considering an incomplete system and that modeling the entire watershed system would resolve this issue. However, this is often not feasible due to finite computing resources. Further, spatially distributed data to properly constrain such models are often not available due to difficulty to access steep mountainous upstream catchments. Thus, an improved understanding of runoff formation in headwater-dominated catchments as isolated systems is necessary to enable their robust modeling. This is important for water resources management, because these systems are often where water is sourced.

A common approach to understand runoff formation is to analyze so-called *aggregated hydrological responses*^19^. For example, catchment hydrographs are commonly analyzed with a top-down approach and decomposed into their individual components—base flow, interflow, overland flow—to understand the system response of the catchmen^19–22^. However, because different combinations of these individual components can generate similar aggregated hydrological responses, this approach yields non-unique descriptions of the catchment system^23,24^.

The analysis of runoff formation can be further constrained through the inclusion of *distributed hydrological responses*, which are point or profile measurements of hydrological state variables and processes inside the catchment^19^. Measured distributed hydrological responses can be used to further constrain the hydrograph analysis by providing valuable local information. Similarly, high-resolution spatio-temporal patterns of hydrological state variables can be obtained through distributed hydrological modeling.

When these patterns are linked to aggregated hydrological responses, they can—at least to some extent—overcome the aforementioned issue of equifinality^17^. In this context, hydrological connectivity is a promising conceptual development^25,26^. Hydrological connectivity describes how different parts of the catchment connect through mass fluxes. The hydrological connectivity of a catchment is typically considered to consist of two parts: (1) the structural connectivity, which is static and can be deduced by catchment characteristics and (2) the dynamic connectivity, which is a transient and emergent property of interactions between rainfall and the catchment^26^. Indices of hydrological connectivity^26–28^ aim to relate these spatio-temporal distributions to hydrological signatures.

In this modelling study, we aim to blue identify the internal hydrological controls on the response of a catchment that is dominated by the dynamics of its headwaters. In particular, we want to understand (1) to what extent surface–subsurface exchange fluxes affect hydrological connectivity and (2) how changes in connectivity are reflected in the catchment’s aggregated hydrograph. For this purpose, we investigate the relationship between aggregated and distributed hydrological response of a headwater-dominated catchment of the East River Watershed, Colorado, USA, which receives significant inflow from its headwater catchments. We use simulation of integrated hydrology processes to highlight the specific set of challenges that are associated with analyzing the hydrological response of a headwater-dominated catchment. We compare the hydrological response of this catchment for different degrees of surface–subsurface exchange, obtained by varying the permeability of the subsurface. Other catchment parameters are kept constant to isolate the effect of surface–subsurface exchange. We explore whether by linking the catchment’s hydrological connectivity to its aggregated discharge, we are able to relate changes in the internal state of the catchment to aggregated hydrological response. This relation may be used to develop a physics-informed systems approach for hydrological applications, where the relation between input and output is directly obtained as an emerging behaviour of the catchment system instead of pure mathematical considerations.

## Materials and methods

### Study site

**Figure 1:**
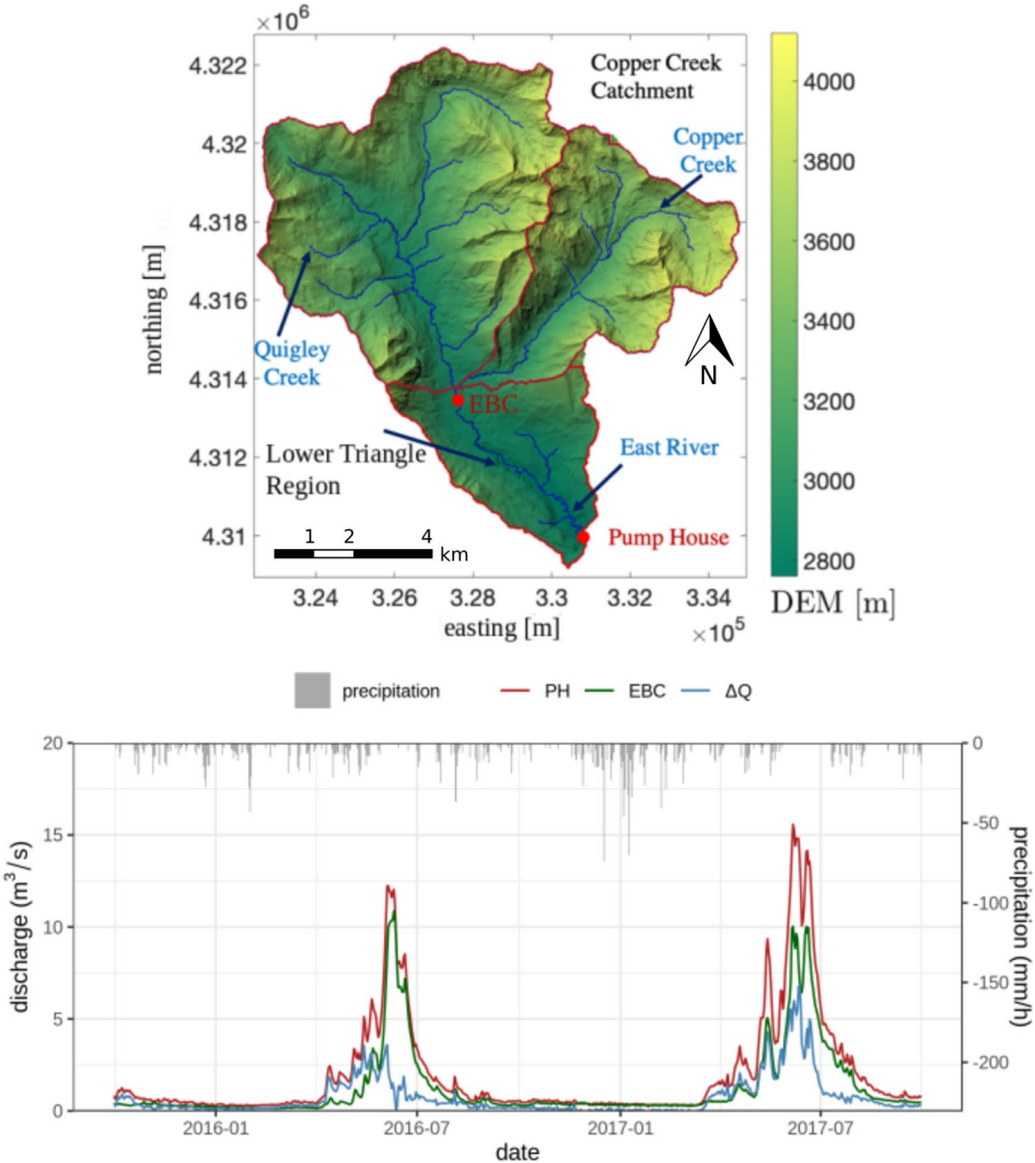
Top: Map of East River Watershed, Colorado, with sub-watersheds (red boundaries). The study region Lower Triangle (LT) is located at the downstream of the watershed. At the outlet of the LT, a pumphouse is located where discharge data is measured daily. The red point labeled with EBC denotes the location of discharge measurements that were used as inflow boundary condition. The map has been generated using the matplotlib library (v3.5)^30^. Bottom: Net flow at the northern boundary (EBC), the discharge measured at the Pumphouse (PH), and difference between the inflow and the outflow, calculated as $\Delta Q = Q_{\text {PH}} - Q_{\text {EBC}}$.

The East River Watershed is a mountainous headwater catchment in the Upper Colorado River Basin, see Fig. 1 (top). As one of two major contributors to the Gunnison River, the East River Watershed is a crucial source of water for the western USA^29^. The majority of this water is stored as snowpack and released over the year. Our study site is the Lower Triangle (LT), a headwater-dominated, 15 km$^2$ subcatchment of the East River Watershed (Fig. 1 (top)).

The geology of the LT is characterized by marine shale of the Mancos formation, with Cenozoic igneous formations intruding the Mancos formation in the western part and the Palaezoic and Mesozoic sedimentary strata in the eastern part, where it forms steeply dipping beds^31^.

The elevation of the LT ranges from 2759 m at its south-western part to 3787 m at its northern part. The lower parts of LT feature a meandering riparian corridor. The stream is fed by high-energy mountain streams from subcatchments on the LT’s north, namely Copper Creek and Quigley Creek^32^—see Fig. 1 (top). The LT is one of the U.S. Department of Energy’s intensively monitored sites to understand the impact of climate change-induced perturbations on watershed systems^29^. A pumphouse (PH), located at the outlet of the LT, extracts water for the municipality of Mt. Crested Butte, Colorado, USA. Daily discharge measurements are provided at this location^33^. In the northern part, the EBC station measures daily discharges in the river, which we use as the inflow hydrograph at the northern boundary of the LT in this work^33^. Discharge is measured with a SonTek Flow Tracker acoustic Doppler velocimeter in conjunction with Solinst Levellogger Edge pressure transducers corrected with barometric pressure^31^.

The hydrology in the region is snow-dominated, due to the continental subarctic climate with long, cold winters and short, cool summers. The annual precipitation ranges between 670 and 1200 mm, where up to 70% is in the form of snow and the majority of the remaining precipitation is in the form of monsoonal rains^31,34^. Snowfall occurs from October through May and monsoonal rains occur from July through September. Snowmelt starts in April and continues through the year. The discharge at the PH station usually peaks in June to July, when snowmelt from the northern subcatchments arrives at the outlet^35^, see also Fig. 1 (bottom).

Figure 1 (bottom) shows the inflow and outflow hydrographs as well as the difference between these hydrographs for the water years 2015 and 2016. In this time interval, the LT contributes about 36% of the total discharge volume at the PH, while the rest is headwater. While the LT contribution to the outflow hydrograph is significant at some times during the high flow periods, it is diminished significantly during the low flow periods. In such a situation, changes in spatial flow dynamics may not always be observable from the hydrograph alone.

### Integrated hydrological solver

We use the *Advanced Terrestrial Simulator* (ATS)^36^—a physically-based, distributed, integrated hydrological model written in C++—to compute the flow and the spatial patterns of different state variables, such as water depth and soil moisture. ATS solves overland flow and subsurface flow on a computational mesh in an integrated manner, using the two-dimensional zero-inertia equation and the three-dimensional Richards(on) equation^37,38^, respectively. The computational mesh describes the topography and subsurface geology and can be a Cartesian grid or an unstructured triangular mesh. Further processes considered in this study are snow storage and evapotranspiration. Snow storage is modeled through a temperature-based approach and evapotranspiration is modeled using the Priestley–Taylor equation^39^.

### Model of the Lower Triangle

#### Model setup

The topography of the catchment is available from a Digital Elevation Model (DEM) with a resolution of 0.5 m^40^. The domain is discretized using a multiresolution, unstructured triangular mesh generated through a wavelet-based meshing strategy^41^. The horizontal resolution is set to 10 m around streams and coarsened up to 160 m in the other parts of the catchment. The vertical resolution goes from 0.1 m at the surface to 30 m in the deeper regions. More information on the mesh is provided in the supplementary information.

Subsurface permeability and soil parameters are assigned according to the geological structure of the subsurface. This structure is predicted by a forward simulation by a three-dimensional geological model, using Emerson-Paradigm’s SKUA-GOCAD software. SKUA-GOCAD uses the proprietary UVT Transform algorithm^42^. The geological model simulation is constrained by USGS geology maps. The geological model results provide a complete high-resolution description of the geology in the domain, including heterogeneity that cannot be obtained by extrapolating the USGS geology maps. Model results indicate that the weathered shale is layered on top of fractured shale and sand. Localized quartz intrusions pinch through the weathered and fractured shale.

Model parameters for the subsurface are taken from the literature. The saturated permeabilities were set based on the values reported by Tokunaga and collaborators^43^, where permeability values at a hillslope in the region are quantified through field observations. The van Genuchten soil parameters^44^ were based on values reported by Xu and collaborators^45^, where a hydro-geochemical model of a neighboring catchment has been calibrated. We summarize permeability and van Genuchten values in Table 1, where the permeability taken from Tokunaga and collaborators^43^ is under the column $K_s$(P0). Here, $\alpha$ is a parameter related to the inverse of the air-entry pressure, *m* is a parameter related to the pore-size distribution, $\theta _r$ is the residual soil water content, and $\theta _s$ is the saturated soil water content. The surface roughness is accounted for by Manning’s law. The Manning coefficient is set to $1.0~\mathrm {sm^{-1/3}}$ throughout the catchment and was not modified for calibration. The model performance is assessed by comparing model results with the measured daily discharge at the PH. Here, we used the hydrographs for the water years 2016 and 2017.

**Table 1:** Permeability values ($K_s$) and van Genuchten soil parameters of the investigated model variations for the geological layers in LT.

Geology	$K_s$ (P0) (m$^2$)	$K_s$ (P−) (m$^2$)	$K_s$ (P$+$) (m$^2$)	$\alpha$ (Pa$^{-1}$)	*m*	$\theta _s$	$\theta _r$
Shallow soil	$9.9\times 10^{-13}$	$1.98\times 10^{-13}$	$4.95\times 10^{-12}$	0.0003	0.6	0.53	0.2
Weathered shale	$1.13\times 10^{-13}$	$2.26\times 10^{-14}$	$5.65\times 10^{-13}$	0.0004	0.5	0.5	0.1
Fractured shale	$1.6\times 10^{-14}$	$3.2\times 10^{-15}$	$8\times 10^{-14}$	0.0004	0.5	0.5	0.1
Quartz	$1\times 10^{-15}$	$2\times 10^{-16}$	$5\times 10^{-15}$	0.0003	0.4	0.67	0.1
Sand	$2.26\times 10^{-12}$	$4.52\times 10^{-13}$	$1.13\times 10^{-11}$	0.0002	0.6	0.2	0.1

Meteorological data needed for the simulation are obtained via the Daymet dataset, which provides daily averaged weather and climatology variables for the entire U.S. at a kilometer resolution^46^. In this work, we use the Daymet estimations for precipitation, temperature, and radiation in the center of the LT and apply them in a spatially uniform manner to the entire catchment. The precipitation is plotted in Fig. 1 (bottom). Temperature and radiation are subject to seasonal cycles (plot omitted). The temperature drops below the freezing point throughout the winter and reaches its peak in the summer. Similarly, radiation decreases during the winter and peaks in summer.

At the north boundary of the domain, we impose a discharge boundary condition to account for the streams entering the domain from this boundary. The boundary condition is informed by measurement data in the stream at the station EBC, which is located close to the north boundary—see Fig. 1 (top). The imposed hydrograph is plotted in Fig. 1 (bottom). At the downstream boundary, a free outflow boundary condition is set.

#### Model variations

We carry out explorative numerical simulations, where we vary the permeability of the subsurface as a way to modulate the surface–subsurface exchange, without changing other catchment characteristics. Thus, changes in the hydrological response can be directly attributed to the surface–subsurface exchange. This is not meant as a thorough sensitivity analysis but as a means to study changes in hydrological response as a consequence of surface–subsurface exchange. For sake of simplicity in the interpretation of simulation results, we did not change the van Genuchten parameters in our model variations. Thus, conceptually, our model variations are not considering different soil types but variations in the saturated permeability of the same soil type. Hereinafter, the model variation P0 corresponds to the model with the parameters reported in Table 1. For the model variation P−, all permeability values are reduced by a factor of 5, while for the model variation P$+$, all permeability values are increased by a factor of 5. The permeability values of all model variations are summarized in Table 1. We note that the reduction and increase by the chosen factor of 5 is rather small when compared to the range of permeability values typically observed in the field. However, as we will see in the following section, even such small changes can yield significantly different runoff behavior.

#### Initial conditions

Initial conditions for the subsequent simulations were generated by a so-called “spin-up” simulation. Starting with initially saturated soil and a dry surface, the domain was drained for 10 years. Saturated soil water content values $\theta _s$ for the subsurface are summarized in Table 1. Afterwards, a constant precipitation with an intensity of 10$^{-8}$ ms$^{-1}$, which corresponds to the annual mean, was applied for 5 years. Finally, a 1 year simulation using the precipitation data for the water year 2016 was carried out to obtain the initial conditions. Throughout the spin-up, the temperature and radiation data from the water years 2016 and 2017 are used.

### Metrics and indices

#### Pearson and Spearman correlation

Pearson’s correlation coefficient^47^ (*r*) between two data sets *x* and *y* is calculated as

1 $$\begin{aligned} r = \frac{\sum _i\left( x_i-\overline{x}\right) \left( y_i-\overline{y}\right) }{\sqrt{\sum _i\left( x_i-\overline{x}\right) ^2}\sqrt{\sum _i\left( y_i-\overline{y}\right) ^2}}, \end{aligned}$$

where $x_i$ and $y_i$ denote the *i*-th entry of the data set *x* and *y*, respectively, and $\overline{x}$ and $\overline{y}$ denote the mean value of the data set *x* and *y*, respectively.

Spearman’s correlation coefficient^48^ ($\rho$) between two data sets *x* and *y* is calculated as

2 $$\begin{aligned} \rho = 1 - \frac{6 \sum _i \left( x_i-y_i\right) ^2}{n\left( n^2-1\right) }, \end{aligned}$$

where *n* is the size of the data sets.

#### Nash–Sutcliffe and Kling–Gupta efficiency

The Nash-Sutcliffe efficiency^49^ ($\textrm{NSE}$) is calculated through

3 $$ {\text{NSE}} = 1 - \frac{{\sum\limits_{t} {\left( {Q_{s} (t) - Q_{o} (t)} \right)^{2} } }}{{\sum\limits_{t} {\left( {Q_{o} (t) - \overline{Q} _{o} (t)} \right)^{2} } }}, $$

where *t* is the time, $Q_s$ is the simulated discharge, $Q_o$ is the observed discharge, and $\overline{Q}_o$ is the mean observed discharge.

The Kling–Gupta efficiency^50^ ($\textrm{KGE}$) is calculated using statistical moments as

4 $$\begin{aligned} \textrm{KGE} = 1 - \sqrt{\left( r - 1 \right) ^2 + \left( \frac{\sigma _s}{\sigma _o} - 1\right) ^2 + \left( \frac{\overline{Q}_s}{\overline{Q}_o} - 1 \right) ^2}, \end{aligned}$$

where *r* is the linear correlation between observation and simulation data, $\sigma _s$ and $\sigma _o$ are the standard deviation in simulation and observation data, respectively, and $\overline{Q}_s$ is the mean the simulation discharge.

In general, higher values of both of these coefficients are considered to suggest a better agreement between simulated and observed hydrographs.

#### Disconnected water clusters as an index of hydrological connectivity

We use the term hydrological connectivity to refer to hydrologically relevant spatial patterns of properties and variables that control flow and transport in a hydrological system^51,52^. In the literature, several indices and measurements of hydrological connectivity have been put forward^26^. One of these indices is the total number of disconnected clusters of ponding water, which is an intuitive metric to quantify hydrological connectivity for surface flow processes^1^. A cluster is defined as one or more “wet” pixels that are geographically separated from the rest of the wet areas. The assumption is that the number of clusters decreases as the hydrological connectivity of the system increases. When the entire system is hydrologically connected, that is to say that all wet areas are connected throughout the domain, the number of clusters is equal to 1.

#### Computation of metrics and indices

Plots in this work have been generated with the matplotlib library^30^ and the tidyverse library^53^. NSE and KGE have been computed with the help of the numpy library^54^. Disconnected water clusters have been counted using the image analysis capabilities of the scipy library^55^.

## Results

### Distributed hydrological response

**Figure 2:**
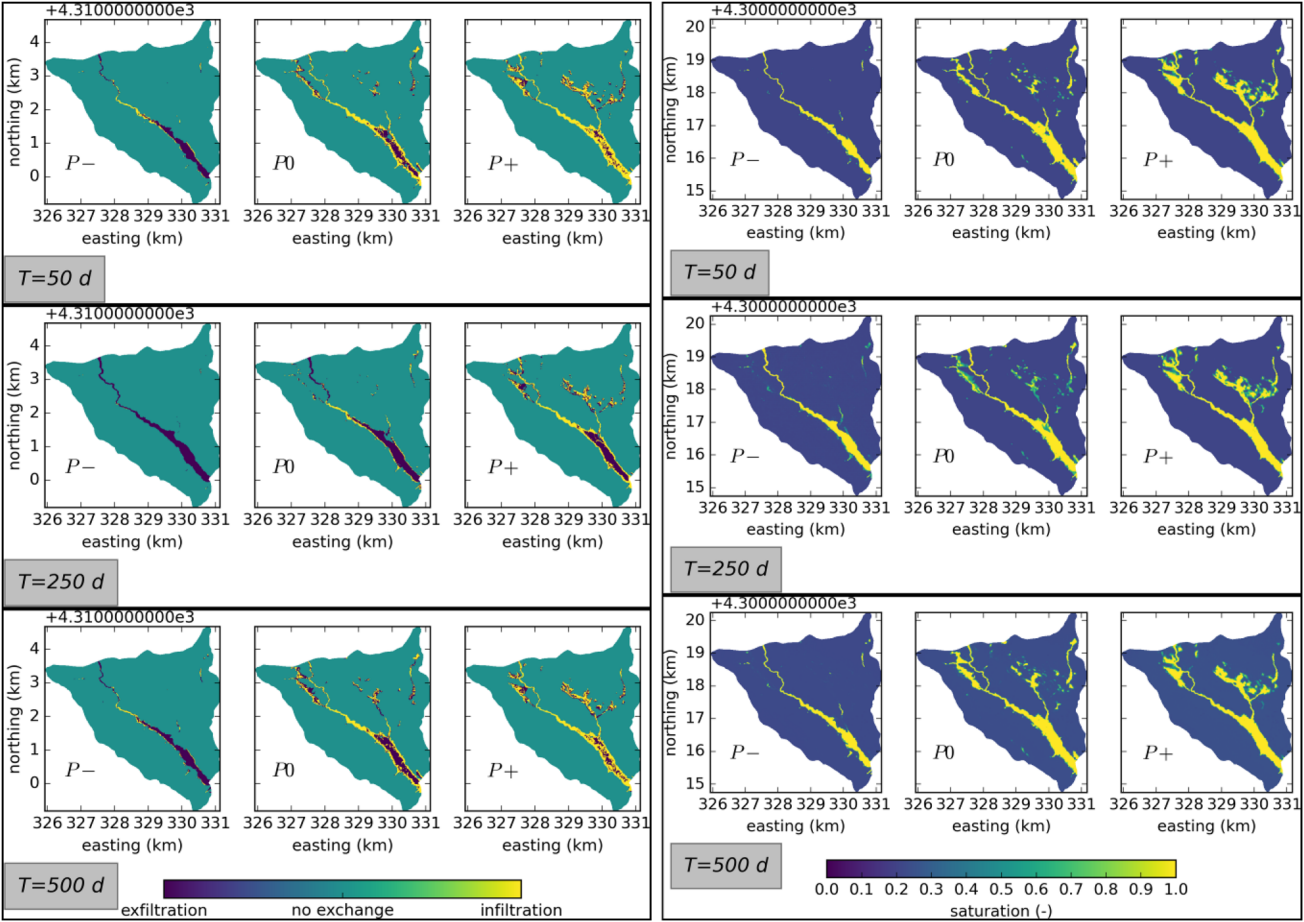
Left: snapshots of the spatial patterns of infiltration and exfiltration at the surface. Right: snapshots of the spatial patterns of soil saturation in the top layer. $T=50$ d: low flow conditions in the first water year, $T=250$ d: rising limb in the first water year, $T=500$ d: low flow conditions in the second water year, shortly before the rising limb.

We quantitatively assess the interaction between surface and subsurface through the exchange mass flux across the surface–subsurface interface. The mass flux is either an infiltration flux, that is to say that water penetrates into the soil from the surface, or an exfiltration flux, which means that soil water is pushed from the subsurface towards the surface due to a pressure gradient. In ATS, negative values of the exchange flux imply exfiltration and positive values imply infiltration. Based on the sign of the exchange flux, spatial patterns of infiltration and exfiltration can be deduced. Figure 2 (left) showcases the differences in these spatial patterns obtained from different model variations at selected time steps. In all cases, water exfiltrates in the riparian areas, especially in the downstream parts, which has flat topograpy and saturated soils—see Fig. 2 (right). This is in agreement with our conceptual understanding of the hydrological cycle, where rainfall infiltrates in the upslope areas, is routed through the subsurface, and exfiltrates in the riparian areas and river channel^15,56^. During low flow conditions ($T = 50$ d and $T = 500$ d, compare also the hydrograph in Fig. 1 (bottom), infiltration is observed in the upstream parts of the river channel. During these times, the soil water in the near surface is lower than during high flow conditions, indicating that the soil is being drained during low flow conditions and therefore has the capacity to absorb water. As the discharge at the outlet begins to increase, the soil in the river channel gets quickly saturated and switches from infiltration to exfiltration. Here, we assume that the simulated time scales are small, such that the influence of deep groundwater is negligible.

Surface–subsurface exchange increases as the permeability of the subsurface is increased. This has significant influence on the spatio-temporal distributions of the exchange flux. As surface–subsurface exchange increases, infiltration and exfiltration rates along ephemeral streams in the northeast of the catchment increase. Water moves faster through the catchment, connecting different compartments on its way. Thus, increased surface–subsurface exchange leads to a higher degree of connectivity inside the catchment.

**Figure 3:**
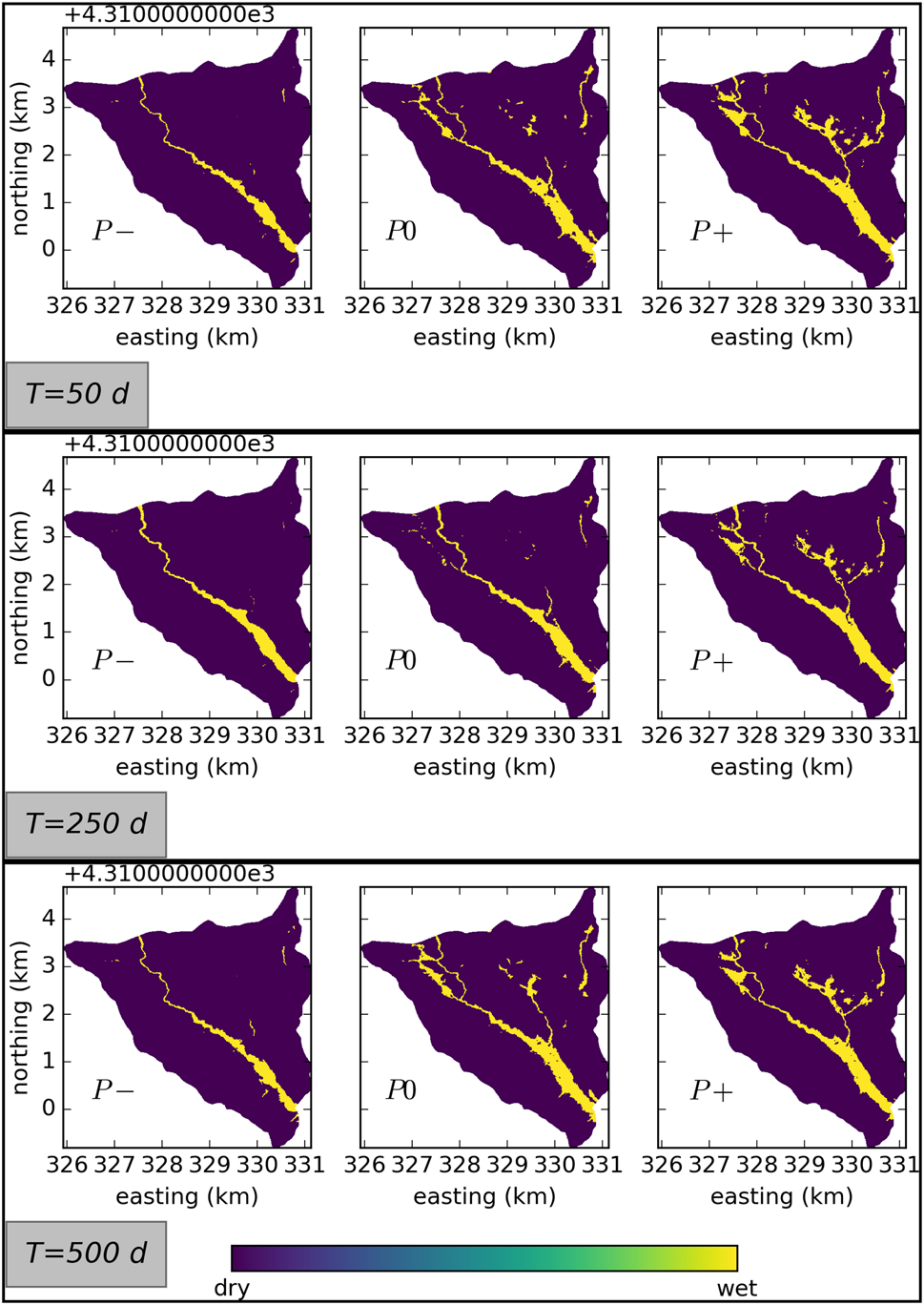
Snapshots of the spatial patterns of wetting and drying at the surface. $T=50$ d: low flow conditions in the first water year, $T=250$ d: rising limb in the first water year, $T=500$ d: low flow conditions in the second water year, shortly before the rising limb.

The degree of surface hydrological connectivity can be investigated by analyzing the spatial distribution of ponding water in the catchment. Maps of the ponding water depths in the catchment can be thresholded into binary wet/dry maps. If the water depth at a certain point is below a certain threshold, the point is considered “dry”, otherwise the point is considered “wet”. Here, we use a threshold of 0.01 m, which is consistent with the literature^1,5^. Figure 3 shows such wet/dry maps for all cases at selected times. Four separate stages of flow regimes are identified in the context of microtopography at the hillslope scale, which can be transferred to the catchment scale to categorize the flow regimes^57^. These flow regime stages are namely (1) local flow, where water flows mostly in disconnected depressions and fills them, (2) channel flow, where local streams start to develop between filled depressions, (3) mixed flow, where filled depressions start to merge as the topography gets inundated, with higher parts remaining dry, and finally, (4) sheet flow, where all depressions are filled and the surface is completely inundated.

The spin-up simulations ensure that at the initial state, the flow regime at the riparian areas reaches sheet flow. In the P− model variation, upslope areas showcase local flow regimes with disconnected puddles that showcase a mixture of in- and exfiltration. The P0 model variation results in localized channel flow regimes in the upslope areas, in combination with local flow regime in parts. The wetland area is extended and in addition to sheet flow showcases mixed flow regimes at its edges. Further increase in surface–subsurface interaction leads to a connection between depressions and leads to a mixed flow regime in the upslope areas, that are then partially connected to the riparian areas through one-dimensional channels. The riparian areas are dominated by sheet flow. Temporally speaking, the low flow conditions ($T = 50$ d and $T = 500$ d) showcase a higher degree of connectivity between the depressions than the conditions at the rising limb ($T = 250$ d), where the ephemeral streams in the upslope areas seem to dry out, reverting the flow regime from channel flow to local flow in these areas. Comparison between $T = 50$ d and $T = 500$ d reveals that the catchment is not fully drained, some of the initially dry localized ponds and ephemeral streams that appear on the rising limb of the hydrograph ($T = 250$ d) persist during the low flow conditions.

### Aggregated hydrological response

**Figure 4:**
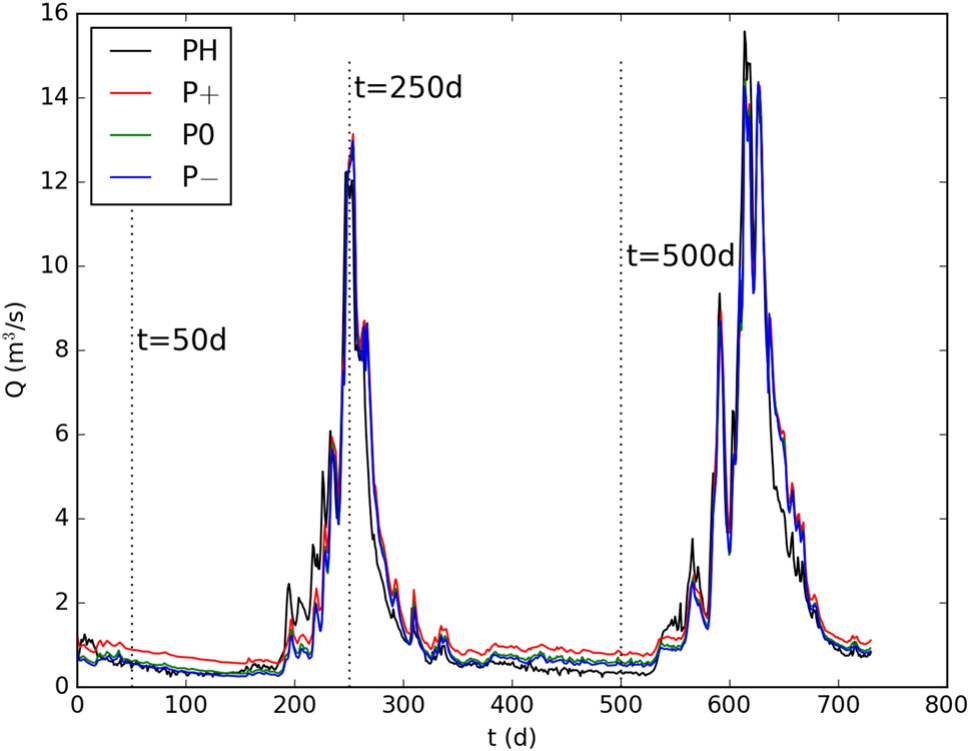
Model discharge predictions at PH for different model variations. Dashed lines indicate $t=50, 250, 500~\textrm{d}$, where snapshots of distributed hydrological response is plotted in Figs. 2 and 3.

**Figure 5:**
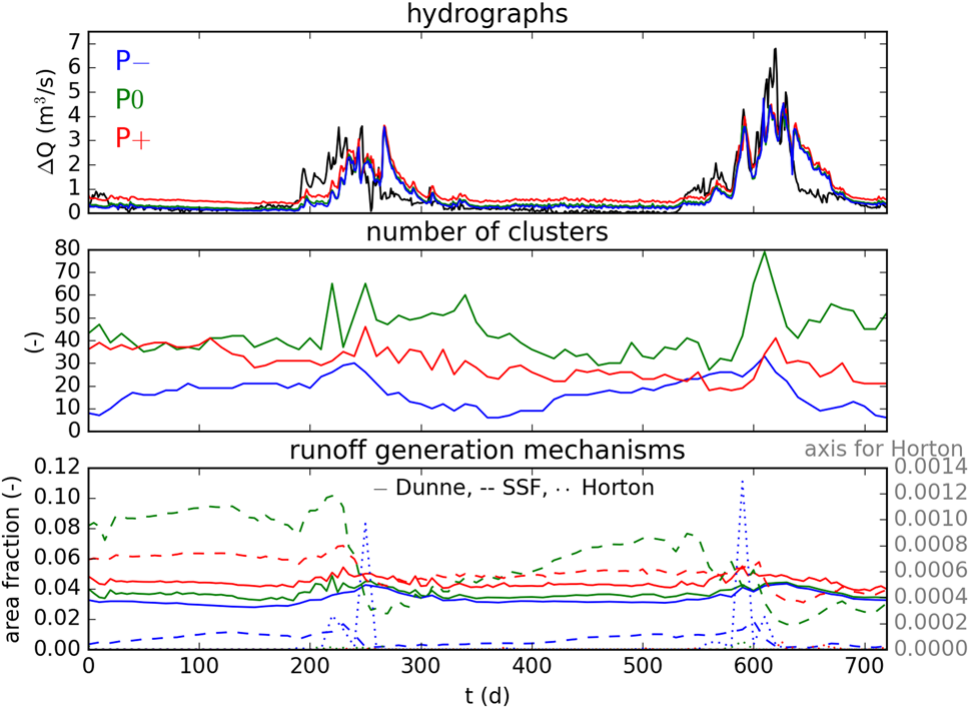
Top: Model discharge predictions at PH for different model variations with the boundary condition subtracted to visualize isolated processes within the catchment. The observed discharge at PH is plotted with a black continuous line. Middle: Number of disconnected wet clusters. Bottom: Runoff generation mechanism dynamics in the catchment. The figure plots the fraction of the catchment area that showcases a certain runoff generation mechanism over time.

We have observed that the different model variations result in different spatial distributions of hydrological variables. We will now proceed to analyze whether these different spatial distributions can be observed in the hydrographs at the outlet of the catchment. Figure 4 compares the computed hydrographs of all model variations and the observed hydrograph (PH). Qualitatively, the hydrographs from all model variatons showcase similar dynamics and accurately capture the peaks. Indeed, the hydrograph produced by P0 is difficult to distinguish visually in this figure. The reason for this might be that during peak flow conditions the soil below the flow paths in the riparian areas quickly becomes saturated, which increases the dependency of the hydrograph on the headwater. This is partially supported by the fact that smaller fluctuations on the rising limb of the first peak are not reproduced, which is one of the rare times when the LT’s contribution to the hydrograph exceeds the contribution of the headwater catchments significantly—see Fig. 1 (bottom). This is similar to the findings by Caviedes-Voullième and collaborators^1^, who observed that for high rainfall intensities, the effect of topography on the hydrograph is reduced, because the surface depression storage fills in the early stages of rainfall onset. Differences in model predictions become more clear when the net catchment hydrograph $\Delta Q_i = Q_i - Q_{\textrm{EBC}}$ is plotted. For each model variation *i*, the net catchment hydrograph is calculated by subtracting the inflow $Q_{\textrm{EBC}}$ from the discharge $Q_i$ computed at the outlet. The net catchment hydrographs are plotted in Fig. 5 (top).

The volume of the discharge is calculated as the integral of the hydrograph, see Table 2. All discharge volumes are in the same order of magnitude. The differences between the discharge volumes are mainly due to discharge differences during low flow conditions, which are especially affected by the change in permeability. The volume of the hydrograph belonging to P$+$ model is the largest, the difference is about $13\%$ of the observed discharge volume. This can be attributed to the increased base flow accumulating throughout the years. The best match for the discharge volume is achieved by the P− model, but the P0 model’s discharge volume does not deviate significantly.

**Table 2:** Summary of discharge volumes, $\textrm{NSE}$ (NS) and $\textrm{KGE}$ (KG) for the observed discharge data and the simulations.

	*V* ($\textrm{m}^3$)	$\Delta V / V_o$ ($\%$)	$\textrm{NS}(Q)$	$\textrm{KG}(Q)$	$\textrm{NS}(\Delta Q)$	$\textrm{KG}(\Delta Q)$
Obs.	$126\times 10^6$	–	–	–	–	–
P+	$142.5\times 10^6$	$+13.1$	0.94	0.86	0.58	0.55
P0	$129\times 10^6$	$+2.5$	0.95	0.96	0.63	0.73
P−	$125\times 10^6$	$-0.8$	0.95	0.97	0.63	0.74

$\Delta V$ denotes the difference in discharge volumes with respect to the observed discharge volume and $V_o$ is the observed discharge volume.

The Nash–Sutcliffe and Kling–Gupta efficiency coefficients are calculated for each simulated hydrograph with respect to the measured time series. The computed coefficients for all model variations are summarized in Table 2, for both the catchment hydrograph *Q* and the net catchment hydrographs $\Delta Q$. The deviation in discharge volumes is reflected in both the $\textrm{NSE}$ and $\textrm{KGE}$ coefficients. While all hydrographs have very similar $\textrm{NSE}$ values, the P$+$ model variation with the highest deviation in the discharge volume yields the lowest $\textrm{NSE}$. Nevertheless, the difference might be considered negligible, even though the base flow in this model variation is overestimated significantly. This is a known limitation of the $\textrm{NSE}$, which tends to place more emphasis on the peaks of the hydrograph^58^. The $\textrm{KGE}$ takes the deviation in base flow into account in a more balanced way. Consequently, the difference in the discharge volumes is better reflected in the corresponding $\textrm{KGE}$ values. P$+$ has the lowest $\textrm{KGE}$ value and the difference is clearly distinguishable. P− has the highest $\textrm{KGE}$ value, followed by P0 by a small margin.

### Linking runoff generation mechanisms to hydrological response

Runoff generation mechanisms are commonly classified as (1) saturation excess (Dunne) runoff, (2) infiltration excess (Hortonian) runoff, and (3) subsurface storm flow^4,59,60^. Using the spatial distributions discussed above, we classify the runoff generation mechanism in a spatially distributed manner. Starting from the wet/dry maps (Fig. 3), we decide for each wet cell, whether the runoff is saturation excess or Hortonian. If the underlying subsurface is fully saturated, the runoff is classified as saturation excess and otherwise it is classified as Hortonian. For dry cells, we classify the runoff as subsurface stormflow if there is flow in the underlying subsurface. These cell-based results can then be aggregated to obtain a single dominant runoff generation mechanism in the domain.

Figure 5 (bottom) compares the temporal evolution of the areal fraction controlled by different runoff generation mechanisms for all model variations. Dunne runoff stays nearly constant during low flow conditions and slightly increases during the hydrographs’s peaks. All model variations result in similar Dunne runoff, especially the P− and P0 model variations. The subsurface stormflow shows variations of a factor of about 80 across the model variations. For all model variations, the subsurface stormflow starts high, rises slightly during the hydrograph’s first rising limb and suddenly drops during the hydrograph’s receding limb. During the low flow period, subsurface flow gradually increases until it drops again during the hydrograph’s second rising limb. The highest subsurface stormflow results from the P0 model, followed by the P$+$ model. The lowest subsurface stormflow results from the P− model. Hortonian flow is only observed during both peaks of the hydrograph. P− model yields the highest Hortonian flow by orders of magnitude. Overall, P0 and P− models result in similar hydrographs, but result from different combinations of runoff generation mechanisms. The subsurface stormflow is inversely related to the surface connectivity in the domain. This is reasonable, since increased subsurface stormflow implies decreased surface runoff, which results in less ponding on the surface.

### Linking permeability of the subsurface to hydrological response

Comparing spatial distributions of permeability with soil saturation reveals that permeability distribution does not significantly affect spatial distributions of soil saturation. In all model variations throughout the subsurface, the Pearson correlation coefficient is the range of $-0.01$ at the top to $-0.13$ at the bottom of the subsurface, indicating no significant linear relationship between these two distributions. Meanwhile, the Spearman correlation coefficient is in the range of $-0.1$ at the top to $-0.4$ at the bottom of the subsurface, indicating that no significant monotonicity can be detected in the relationship, that is to say, locally higher permeability does not lead to locally higher saturation. This indicates that in our case, the topography and surface hydrology are the dominant control on the spatio-temporal distribution of soil saturation throughout the subsurface. In general, the correlations are slightly worse for the P− model variation, where the surface–subsurface interaction is decreased.

### Linking hydrological connectivity to hydrological response

#### Surface connectivity

The wet/dry maps in Fig. 3 show disconnected clusters of wet cells. When precipitation or the inflow increase, the flow regime in the catchment shifts from local flow towards sheet flow—that is to say, it shifts from water flowing mostly in disconnected depressions to filled depressions and inundated surfaces^57^. This shift in the flow regime is marked by a decrease in the number of disconnected clusters. Consequently, a decrease in the number of disconnected clusters implies an increase in the degree of connectivity and vice versa. The evolution of the number of these disconnected clusters is plotted in Fig. 5 (middle). The rainfall does not affect the connectivity in the catchment, which is in contrast to the findings by Han and collaborators^61^, where rainfall characteristics were found to dominate connectivity in a headwater catchment. This difference is most likely due to the LT being headwater-dominated. The connectivity of the catchment decreases during the rising limb of the hydrographs and gradually increases in the recession phase. This is roughly the behavior reported by Khosh Bin Ghomash and collaborators^5^, where the decrease in connectivity is related to the microtopography getting partially inundated. Similarly, in the LT the wetland area extends as the hydrograph rises, causing local flow with disconnected clusters of water at its edges. Increasing the surface–subsurface exchange alters the connectivity in the catchment but the relationship is non-linear. The P0 model variation results in the least degree of connectivity, and increasing and decreasing the surface–subsurface exchange both results in an increased connectivity.

**Figure 6:**
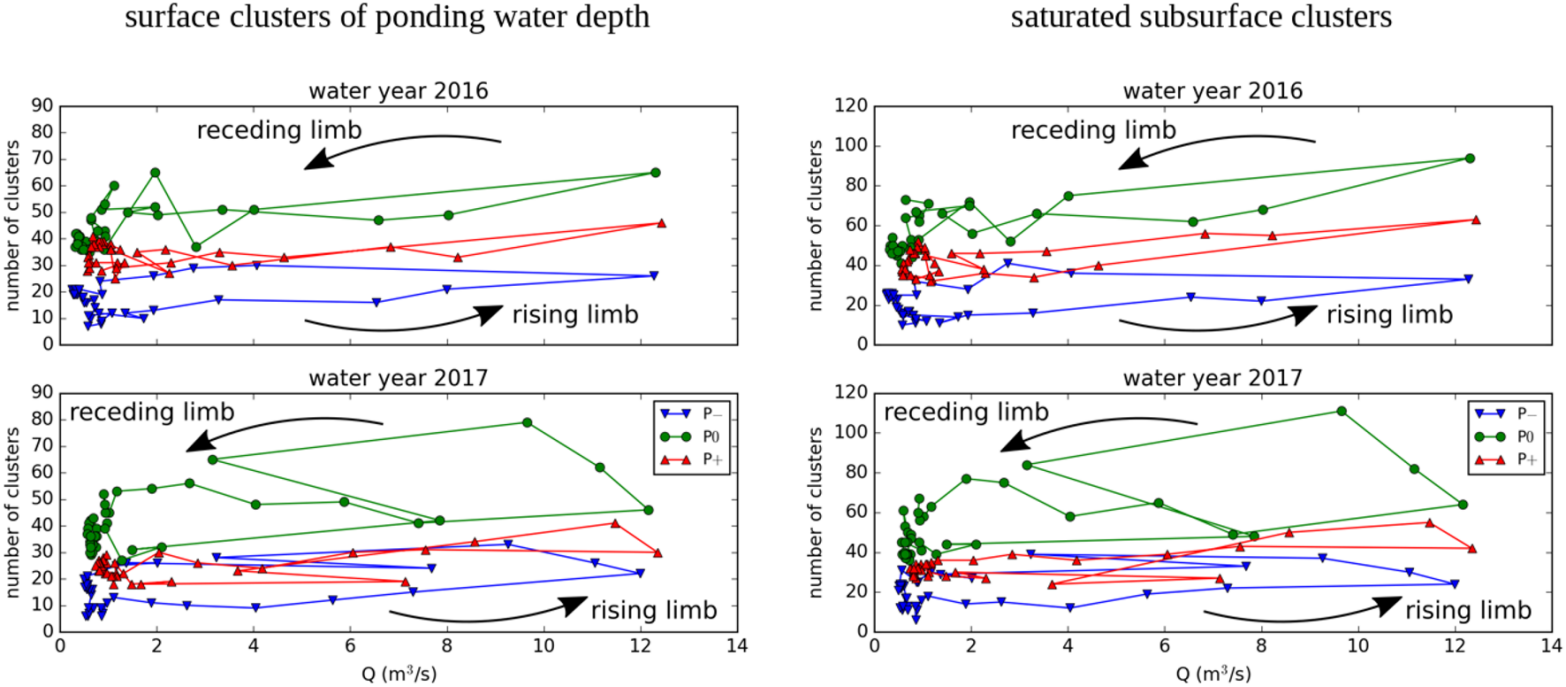
Relationship between number of disconnected wet clusters and discharge at the outlet for both simulated water years. Left: surface clusters of ponding water depth, right: saturated subsurface clusters.

Figure 6 (left) shows a hysteretic relationship between the number of disconnected wet clusters plotted and the discharge at the outlet. As the discharge at the outlet increases, the number of disconnected clusters increases. This indicates that the degree of connectivity in the catchment decreases, reaching its minimum on the receding limbs of the hydrograph. This marks the transition of the flow regime in the catchment from sheet flow back to local flow. We observe a thresholding behavior at about 2 m$^3$/s, after which the surface connectivity does not decrease significantly. However, in the second water year, the P0 model variation showcases a rapid disconnection of the catchment during the receding limb of the hydrograph, leading to pronounced new maxima in the disconnected wet cluster number.

#### Subsurface connectivity

Similar to the thresholding to create wet/dry distributions, the subsurface saturation can be thresholded into a binary saturated/unsaturated distribution. Similar to the wet clusters on the surface, such saturated cluster distributions can be used to derive metrics of subsurface connectivity^15,28^. Figure 6 (right) shows the number of disconnected saturated clusters at the top subsurface layer and its relation to the discharge at the outlet of the domain. The subsurface connectivity follows the same dynamics as the surface connectivity. The subsurface displays a higher degree of connectivity during low flow conditions and disconnects as the hydrograph rises and falls. This might be related to the widening of the wetland area, which inundates previously dry areas and causes localized saturated clusters. We identify the same threshold of 2 m$^3$/s, after which the connectivity for most model variations does not decrease significantly.

## Discussion

In order to understand the effect of surface–subsurface exchange on distributed and aggregated hydrological response, we explore whether these two responses can be linked through the concept of hydrological connectivity.

Surface–subsurface exchange causes complex spatial runoff and connectivity dynamics at the catchment scale, which may not be observable through aggregated hydrological signatures. Indeed, the surface–subsurface exchange alters the catchment behavior—as observed both in the connectivity and the localized runoff generation mechanisms—in a non-linear manner that still results in similar hydrographs. The different initial conditions that result from the individual spin-up simulations carried out for each model variation amplify this non-linearity^62^. Here, the surface topography was identified as the dominant control on spatio-temporal distributions of hydrological variables both at the surface and in the subsurface.

In headwater-dominated catchments, we show that the catchment’s own signal may be overshadowed by the headwater dynamics. In this study, we assume that catchment characteristics only change in the downstream catchment, but not in the headwater catchment—the inflow boundary condition remains the same. In reality, such changes may also apply to the headwater catchment, which would affect the inflow boundary condition and consequently, would influence our results. Still, we expect the effect of the catchment characteristics on aggregated signatures to diminish as the volume of the headwater inflow increases.

Both surface and subsurface connectivity dynamics follow the same pattern, see Fig. 6 (left) and (right). High connectivity during low flow conditions, gradual disconnectivity until a threshold whereinafter either no significant change or rapid increase in disconnectivity is observed. The thresholding behavior is consistent with the results by Weill and collaborators^15^, but the relationship between connectivity and discharge is the opposite—the highest degree of connectivity is reached during peak flow. We speculate that this might be because the catchment investigated by Weill and collaborators^15^ is a headwater catchment, while our study considers a catchment with headwater inflow, however more thorough studies are necessary to make a conclusive statement.

The differences in the connectivity dynamics between the model variations do not clearly manifest in the hydrographs, see the overlapping hydrographs in Fig. 4. Differences in the rising limbs of the hydrographs from different model variations, which have been reported for microtopographical controls on runoff^1,5^, have not been observed. This indicates that the rising limb of the hydrograph is strongly related to the structural connectivity of the topography, which has been kept constant in this study. However, the inflow from the headwater catchments and the LT runoff are entangled to an extent that makes it difficult to identify the individual contributions, as seen in Fig. 1 (bottom). This inability to distinguish between individual contributions is a particular challenge in headwater-dominated catchments. From simulation snapshots in Fig. 3, we observe that the runoff in the LT leads to the occurrence of ephemeral streams in the domain that feed the river valley for a brief period of time. To what extent these contributions generate inundations in the river valley remains to be ellucidated.

We see in Table 2 that the change in the distributed hydrological response triggered by a change in surface–subsurface exchange fluxes is reflected in the difference in discharge volumes as well as the $\textrm{KGE}$. The $\textrm{NSE}$ is not sufficient to detect these changes. This suggests that the most significant impact of surface–subsurface exchange on the aggregated hydrograph is the rainfall partitioning into overland flow and subsurface flow during low flow conditions, which is better reflected in the discharge volumes and the $\textrm{KGE}$. The discrepancies between computational and observational data were drowned out by the signal of the inflow from the headwater catchments. A reason for the relatively good fit of the aggregated hydrograph among all model variations is that although watershed parameters such as permeability and roughness are important, hydrographs at the outlet are largely controlled by topography and shape of the watershed. Thus, with high-resolution topography data, the ATS model is able to reproduce the hydrograph at the outlet reasonably well. This has been demonstrated across multiple catchments^63^. The good fit might further result from the fact that the catchment response is heavily affected by the inflow boundary conditions.

The net catchment hydrographs ($\Delta Q$) yield lower $\textrm{NSE}$ and $\textrm{KGE}$ values than those from the aggregated hydrographs (*Q*), indicating that $\Delta Q$ is not captured as accurately. In part, this poorer fit may be explained by the fact that $\Delta Q$ neglects the temporal delay between the inflow and the outflow. But it also indicates that the current model does not completely capture all catchment processes correctly and $\Delta Q$ is more sensitive to this. Ultimately, it emphasizes the fact that the headwater inflow overshadows the contribution of the catchment in the aggregated hydrograph. Thus, when the model is calibrated to the aggregated hydrograph, the net catchment contribution might not be captured accurately. This mismatch can only be detected when the net $\Delta Q$ is available as is the case in this study. Point measurements inside the catchment that inform about the distributed hydrological response would help to further constrain the model.

An effect of varying the permeability is that the volume of the hydrograph is different in each scenario, accounted for mostly by changes in the unsaturated zone. The effect on the distributed hydrological response is complex, because these changes in the unsaturated zone storage behavior are amplified by topographical drivers and increase the local heterogeneity—new locations of in- and exfiltration are created, see Fig. 2. In addition, as seen in Fig. 3, water ponds at different locations, indicating also a change in the surface storage. This can be observed in the surface connectivity of the catchment, shown in Fig. 6, where different degrees of connectivity are obtained for different model variations.

A limitation of this study is that only three model variations with a relatively narrow permeability range have been compared. In order to gain a more complete understanding of the effect of surface–subsurface exchange on the hydrological response, a systematic study spanning a wider range of permeability values is needed to detect possible thresholding behavior in the catchment’s response. Further, permeability values were varied in bulk, which does not result in new patterns of heterogeneity. The effect of changing the permeability of individual layers was not explored. Another limitation is the spatially uniform precipitation that has been used for the studies, which does not allow the study of localized precipitation events. With elevation that spans more than 1200 m across the LT, orographic effects may enhance such localized precipitation events and thus, the precipitation within the catchment may show high variability that is not accounted for in this study. The importance of spatially heterogenous precipitation on catchment response has been reported by Shuai and collaborators^63^.

## Conclusions

Using computational experiments, we investigated the effect of surface-subsurface exchange on distributed and aggregated hydrological response in a headwater-dominated catchment. Based on our results, we conclude that (1) surface–subsurface exchange significantly affects hydrological connectivity in catchments not only by runoff partitioning but also by creating new spatial patterns of in- and exfiltration. In the presence of significant headwater inflow, surface–subsurface exchange controls the in- and exfiltration dynamics in the riparian areas. We further conclude that (2) distinct states of hydrological connectivity cannot be directly observed in the catchment’s aggregated hydrograph, if it is dominated by headwater inflow. However, linking indices of connectivity such as disconnected cluster count to the aggregated signature reveals hysteretic behavior, indicating that the system’s history remains relevant for the aggregated hydrological response even in headwater-dominated catchments. From the investigated model variations, the P0 model seems to be the most reliable, because its parameters have been taken from other studies in the region and the $\textrm{NSE}$ and $\textrm{KGE}$ values are acceptable when compared to the other model variations.

The temporal evolution of connectivity in the headwater-dominated catchment is the opposite of what has been reported for a headwater catchment^15^. This may be related to the widening of the riparian areas due to increased headwater inflow during peak flow conditions, which inundates previously dry areas and creates disconnected local flow. This widening of the riparian areas may not be as significant in headwater catchments, where the emergence of ephemeral streams connecting disconnected clusters dominates the connectivity. The inverted temporal evolution implies that geochemical hotspots associated with hydrological (dis)connectivity are different in headwater-dominated catchments compared to catchments without inflow from their upstream. It would be desirable to see if this behavior is generalizable to other catchments.

We have shown that the number of disconnected water clusters is a straight-forward index of connectivity in this study. However, generally, this index does not make use of other available information about the catchment’s state or complexity. Normalizing the index by the wet surface area, for example, may potentially help to distinguish between different catchment states. Such a normalized index would behave differently for the beginning of a rainfall event—wet surface area is small but extending—and the end of the rainfall event—wet surface area is large but decreasing. Shannon’s entropy index^64^ is an alternative index that gives a measure of inner complexity of a system, including flow complexity^28^ and could be linked to aggregated signatures for additional insights. Linking the catchment’s hydrological connectivity to its aggregated discharge could be used to develop physics-informed systems approaches that account for the hydrological system’s internal state more accurately than current approaches. The dynamics of a suitable index of hydrological connectivity could then be used to define the relation between input and output of the system. While beyond the scope of this work, this would allow to conceptually account for spatially distributed hydrological response in so-called lumped hydrological models.

In terms of hydrograph analysis, the KGE is found to detect the discrepancy in the base flow better than the NSE, which is in agreement with the literature^58^. Depending on the aim of the study, it may be seen as beneficial that changes in connectivity do not affect the aggregated hydrograph, however, the correct representation of connectivity may be significant when modelling morphodynamics, ecological processes, or (bio)geochemistry^9,11,65–67^. A promising development in linking distributed and aggregated hydrological responses is the use of stable water isotope-based modeling approaches^68–70^, which allow constructing a more precise relationship between spatiotemporal distributions and the hydrograph that can be directly supported by field measurements^71,72^.

## Supplementary Information

Supplementary Information.

## Data Availability

All data used in this study is available through ESS-DIVE at https://data.ess-dive.lbl.gov, under permissive licenses. The digital elevation model is available at https://doi.org/10.21952/WTR/1412542 and the discharge data is available at https://doi.org/10.21952/WTR/1495380.

## Code availability

The Advanced Terrestrial Simulator is available through Github, at https://github.com/amanzi/ats/releases, under a 3-clause BSD license. ATS Release 1.0.0, used in this study, was tagged on May 14, 2021. All model results are archived in Zenodo, DOI: https://doi.org/10.5281/zenodo.7097093.
