# Supplementary material for: Understanding the hydrological response of a headwater-dominated catchment by analysis of distributed surface–subsurface interactions
Source: Sci Rep. 2023 Mar 22;13:4669. doi: 10.1038/s41598-023-31925-w (PMC10033882; doi:10.1038/s41598-023-31925-w)
Supplement: Supplementary file 1 — Supplementary Information. [file 41598_2023_31925_MOESM1_ESM.pdf]

Supplementary Information to “Understanding the hydrological response of a headwater-dominated catchment by analysis of distributed surface–subsurface interactions”

Ilhan Özgen-Xian[1,2,‡], Sergi Molins[1], Rachel M. Johnson[1], Zexuan Xu[1], Dipankar Dwivedi[1], Ralf Loritz[3], Utkarsh Mital[1], Craig Ulrich[1], Qina Yan[1], and Carl I. Steefel[1]

[1]Earth and Environmental Sciences Area, Lawrence Berkeley National Laboratory, USA
[2]Institute of Geoecology, Technische Universität Braunschweig, Germany
[3]Institute of Water and River Basin Management, Karlsruhe Institute of Technology, Germany
[‡]i.oezgen@tu-braunschweig.de

ABSTRACT

In this document we provide supplementary information on the computational mesh used in the main document.

1 Computational mesh and mapping of geology

The computational mesh is generated through a wavelet-based meshing approach[1]. We decide the horizontal resolution by computing an idealized, stationary flow field with the D∞ algorithm[2]. In this algorithm, the water in each cell flows along the steepest topographic gradient into one or two adjacent cells in the Moore neighborhood. This cellular automaton essentially predicts a steady state flow field that results from unceasing rainfall, and thus, predicts flow paths for a fully connected catchment. This flow field is used as input data to carry out a Haar wavelet analysis of this flow metric raster to predict a resolution level at each raster point based on a specific threshold ε. The multiresolution mesh must explicitly resolve all spatial variations in the data above ε, but can coarsely resolve regions with variations below ε. Consequently, low values of ε lead the wavelet analysis to predict higher resolution levels. Increasing ε leads to the prediction of lower resolution levels. Once the resolution levels are predicted, the multiresolution mesh is generated by starting with an initial uniform resolution mesh, which is then recursively refined around each raster point as many times as the predicted resolution level at this point. This procedure gives a multiresolution unstructured triangular mesh, which is then extruded in the vertical direction using 22 layers. The vertical resolution of the mesh from top to bottom is given in Table 1. In total, the mesh is 100 m deep. The resulting mesh—shown in Figure 1 (left)—contains 894,740 cells. The code to generate the mesh was tagged with v0.3 on September 28, 2022, and is available under the GNU General Public License v3.0 at: https://git.rz.tu-bs.de/i.oezgen/wamr2d

The geology predicted by this model is mapped onto the computational mesh using a nearest neighbor interpolation, see Figure 1 (right). Due to the interpolation, subgrid-scale heterogeniety and geological boundaries may not be captured accurately in the mesh. The near surface region, where we expect the most relevant transient processes to occur, is vertically discretized with a fine resolution—0.1 m to 5 m—and is expected to be less affected by such inaccuracies. The effect of the interpolation may become more pronounced in the deeper surface, where vertical resolutions up to 30 m are used.

References

1. Özgen-Xian, I. *et al.* Wavelet-based local mesh refinement for rainfall-runoff simulations. *J. Hydroinformatics* **22**, 1059–1077 (2020).

2. Tarboton, D. G. A new method for the determination of flow directions and upslope areas in grid digital elevation models. *Water Resour. Res.* **33**, 309–319 (1997).

Layer	Resolution (m)
1	0.1
2	0.1
3	0.1
4	0.2
5	0.5
6	1.0
7	1.0
8	1.0
9	1.0
10	1.0
11	1.0
12	2.0
13	3.0
14	3.0
15	3.0
16	3.0
17	3.0
18	3.0
19	3.0
20	10.0
21	30.0
22	30.0

Table 1. Vertical resolution from top to bottom, Layer 1 is near the surface and Layer 22 is at the bottom of the mesh.

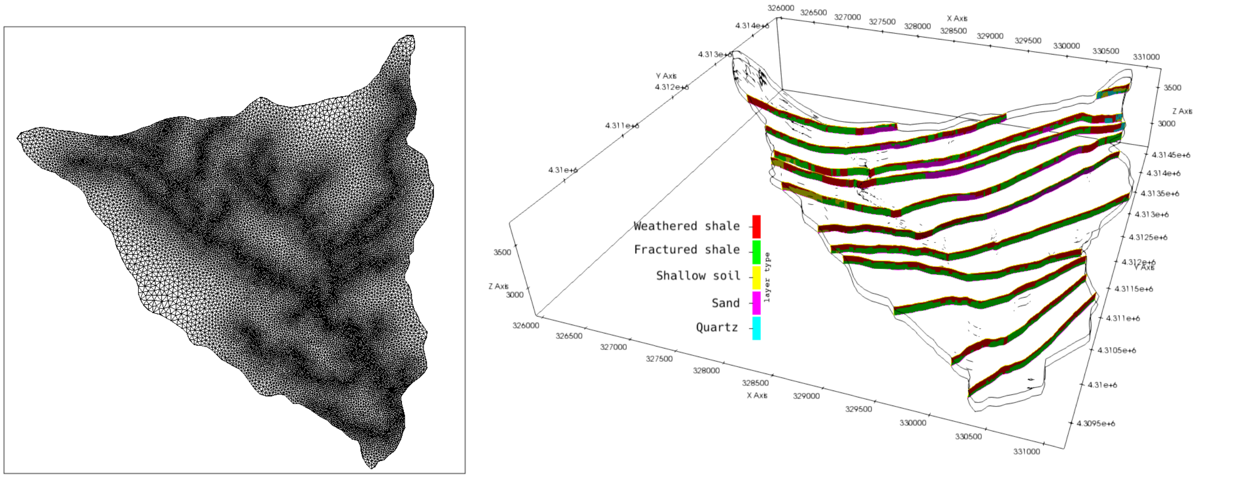

Figure 1. Top view of the computational mesh (left) and a three-dimensional view of selected cross sections showing the geological layer structure in the LT as interpolated onto the computational mesh (right)
